# Influence of radiographic projection and patient positioning on shortening of the fractured clavicle

**DOI:** 10.1016/j.jseint.2020.03.005

**Published:** 2020-05-18

**Authors:** Paul Hoogervorst, Arnoud van Geene, Udo Gundlach, Abel Wei, Nico Verdonschot, Gerjon Hannink

**Affiliations:** a Department of Orthopaedic Surgery, Radboud University Medical Center Nijmegen, Nijmegen, The Netherlands; b Department of Orthopaedic Surgery, University of Minnesota, Minneapolis, MN, USA; c Department of Orthopaedic Surgery, Isala Zwolle, Zwolle, The Netherlands; d Department of Orthopaedic Surgery, Admiraal De Ruyter Ziekenhuis, Goes, The Netherlands; e Emergency Department, Radboud University Medical Center Nijmegen, Nijmegen, The Netherlands; f Department of Biomechanical Engineering, University of Twente, Enschede, The Netherlands; g Department of Operating Rooms, Radboud University Medical Center Nijmegen, Nijmegen, The Netherlands

**Keywords:** Clavicle, fractures, radiological imaging, shortening, displacement, interrater agreement, intrarater agreement

## Abstract

**Background:**

Radiographic measurements of shortening and vertical displacement in the fractured clavicle are subject to a variety of factors such as patient positioning and projection. The aims of this study were (1) to quantify differences in shortening and vertical displacement in varying patient positions and X-ray projections, (2) to identify the view and patient positioning indicating the largest amount of shortening and vertical displacement, and (3) to identify and quantify the inter- and intraobserver agreement.

**Methods:**

A prospective clinical measurement study of 22 acute Robinson type 2B1 clavicle fractures was performed. Each patient underwent 8 consecutive standardized and calibrated X-rays in 1 setting.

**Results:**

In the upright patient position, the difference of absolute shortening was 4.5 mm (95% confidence interval [CI]: 3.0-5.9, *P* < .0001) larger than in the supine patient position. For vertical displacement, the odds of being scored a category higher in the upright patient position were 4.7 (95% CI: 2.2-9.8) times as large as the odds of being scored a category higher in supine position. The odds of being scored a category higher on the caudocranial projection were 5.9 (95% CI: 2.8-12.6) times as large as the odds of being scored a category higher on the craniocaudal projection.

**Conclusion:**

Absolute shortening, relative shortening, and vertical displacement were found to be the greatest in the upright patient positioning with the arm protracted orientation on a 15° caudocranial projection. No statistically significant differences were found for a change in position of the arm between neutral and protracted.

Radiographic measurements of shortening and vertical displacement in the fractured clavicle are subject to a variety of factors such as patient positioning,^3^^,^^23^^,^^26^ point in time after trauma,^26^^,^^27^ anatomic side-to-side difference,^6^^,^^14^ and projection.^2^^,^^30^^,^^32^ Combined with the sigmoid shape of the clavicle in 2 planes, adequate and reliable measurements of the shortening and vertical displacement on a 2-dimensional radiographic image are challenging. All the above-mentioned factors can lead to differences in measured results and thus varying degrees of shortening and vertical displacement that subsequently could influence the choice of treatment.^16^

In spite of this knowledge, there is no universal and standardized protocol that is being used throughout the body of literature to obtain comparable results. Methods used to assess shortening include clinical evaluation using a tape measure^24^ and radiographic evaluation by means of a tilted anteroposterior views of the clavicle (ranging from a 45° craniocaudal to 45° caudocranial views),^1^^,^^7^^,^^10^^,^^22^^,^^33^^,^^34^ anteroposterior panoramic views,^9^^,^^13^^,^^19^^,^^21^^,^^29^ tilted posteroanterior views,^30^ computed tomography scans,^11^ or the method used is not reported.^8^

There is increasing evidence supporting surgical management of displaced, shortened, and/or comminuted clavicle fractures because of lower rates of non- and malunions as well as an earlier functional return and increased patient satisfaction.^4^^,^^17^^,^^20^^,^^25^^,^^35^^,^^36^^,^^38^

There are contradictory reports on the importance of shortening as a relative indicator for surgery. Some studies report that the shortening of 15-20 mm or >8.9% is a predictor of a worse union rates and functional outcomes when treated conservatively.^7^^,^^8^^,^^13^^,^^19^^,^^21^^,^^22^^,^^24^^,^^28^^,^^34^ Others report no association between shortening and functional outcome.^9^^,^^11^^,^^29^ A survey study among upper extremity surgeons reported that 60% use shortening as the most important factor in the decision for surgical vs. nonsurgical treatment.^18^

A previous study by our group showed differences in measurements of shortening of up to 6.0 mm between different projections on digitally reconstructed radiographs (DRRs) of the same fractured clavicle.^16^ To our knowledge, no studies have been performed that evaluated the extent of these differences using proper X-ray images.

The aims of this study were (1) to quantify the difference in measurements of shortening and vertical displacement by using a standardized method of measuring displaced midshaft clavicle fractures in varying patient positions (supine vs. upright and arm in neutral vs. protracted position) and direction of the X-ray beam (15° caudocranial vs. 15° craniocaudal) in absolute and relative measures, (2) to identify the view and patient positioning indicating the largest amount of shortening and vertical displacement, and (3) to identify and quantify the differences in inter- and intraobserver agreement between these variables.

## Materials and methods

A prospective clinical measurement study quantifying the influence of patient positioning and X-ray direction on the measurement of shortening and vertical displacement of the fractured clavicle was conducted in 2 Dutch hospitals (Radboud UMC and AdRZ) between May 2016 and November 2017. Informed consent was obtained from all participants.

All patients aged ≥18 years with an acute Robinson type 2B1 clavicle fracture were asked to participate. Patients with multiple traumas, intoxication, inability to follow instruction, pathological fractures, or soft tissue damage were excluded.

In order to evaluate the influence of patient positioning (supine vs. upright and arm in neutral vs. protracted position) and the influence of projection (15° caudocranial vs. 15° craniocaudal), each patient underwent 8 consecutive standardized and calibrated X-rays in 1 setting after administration of sufficient analgesics. All possible combinations of the 3 evaluated variables were included (Supplementary Appendix S1).

The protracted positioning of the arm, which would occur if the X-ray image were taken with the arm in a sling or collar and cuff, was simulated by placing the hand of the affected side on the contralateral anterior superior iliac spine. To measure differences between X-ray projections, the 15° caudocranial and 15° craniocaudal were used. Earlier research on this topic found that the difference between these views of 2.0 mm was the smallest that was statistically significant.^16^ It was assumed that if these views show statistically significant differences in this study, the differences between 30° caudocranial and all other views would be statistically significant as well. Additional views were omitted to minimize radiation exposure to a minimum.

**Figure 1:**
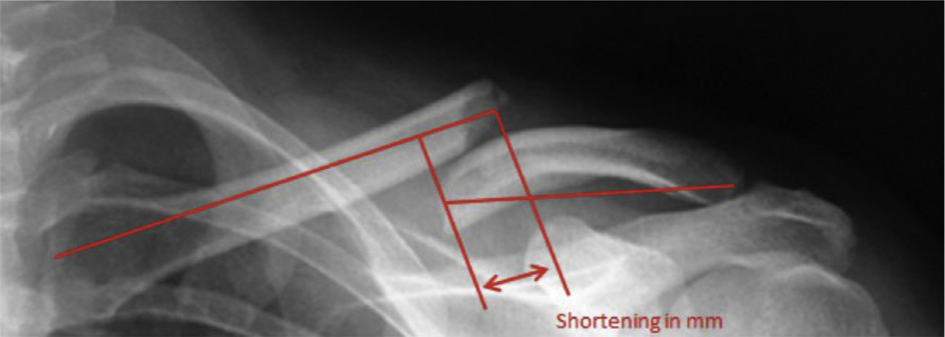
Standardized method of measuring the shortening of the midshaft clavicle fracture as adapted from Silva et al.^31^

A standardized method for measuring shortening as described by Silva et al^31^ was used (Fig. 1). This methodology and the precise definition of the reference points were discussed and agreed upon by the observers. In short, lines through both the medial and lateral fragment of the clavicle were drawn from the center of the acromioclavicular or sternoclavicular joint to the center of the fracture plane. The lengths of these lines represent the lengths of the fragments. Next, a perpendicular line was drawn from the line through the medial fragment at the fracture plane. Subsequently, a parallel line was drawn to this line at the point where the line through the lateral fragment intersects the fracture plane. The difference between the latter 2 lines indicates the amount of shortening in millimeters (mm). Relative shortening was calculated by dividing the shortening in mm by the sum of the length of the medial and lateral fragments in mm × 100. Displacement was documented by allocating it to 1 of 3 categories (0%-50%, 50%-100%, or >100%). The authors did not compare the fractured side with the contralateral side because the existing anatomic side-to-side difference of ≥5 mm in 30% in the population would introduce additional margins for error.^6^^,^^14^

Two observers (2 orthopedic surgeons PH and AG) evaluated the images for each patient in random order. In order to calculate intraobserver agreement, one of the observers (PH) performed a second evaluation of the same images 2-4 weeks after the first measurements were performed. Measurements were performed using the hospitals IMPAX software (version 6.5.3.1005).

Descriptive statistics were used to summarize the data. Intraclass correlation coefficients (ICCs) were used to assess the intra- and interobserver agreements for each of the projections and patient positions for numerical data. Intra- and interobserver agreements for the categorical data concerning vertical displacement classification were reported using Gwet's AC1.^12^ Gwet's AC1 was used as an alternative to Cohen's kappa, because it provides a chance-corrected agreement coefficient, which is better in line with the percentage level of agreement and less sensitive to prevalence and symmetry compared with Cohen's kappa.^12^^,^^37^ ICCs and Gwet's AC1 were interpreted as follows: <0.40 poor; 0.40-0.59 fair; 0.60-0.74 good, 0.75-1.00 excellent.^5^ The ICC was calculated from a 2-way random-effects model, for absolute agreement.

Linear and ordinal mixed models were used to study the effect of patient position, arm position, and X-ray projection on shortening and displacement, respectively. Patient position (upright/supine), arm position (neutral/protracted), and X-ray projection (15° craniocaudal/15° caudocranial) were used as fixed factors. Patient ID was used as a random factor. Statistical analyses were performed using R version 3.6.0 (R Foundation, Vienna, Austria). *P*-values <.05 were considered statistically significant.

## Results

Twenty-four patients with Robinson type 2B1 clavicle fractures were included, and for all patients, the imaging protocol was completed. Two patients did not have calibrated images, leaving 22 patients (21 male, 1 female) available for analysis. Fracture laterality was equally distributed (11 right, 11 left). The average age of the participants was 46.7 years (standard deviation [SD], 15.8; range, 19-74 years).

The intraobserver measurements of absolute shortening (0.91, 95% confidence interval [CI]: 0.87-0.93), relative shortening (0.92, 95% CI: 0.89-0.94), and vertical displacement (0.77, 95% CI: 0.69-0.85) were excellent (Table I). The interobserver measurements of absolute shortening (0.67, 95% CI: 0.50-0.77), relative shortening (0.72, 95% CI: 0.56-0.81), and vertical displacement (0.67, 95% CI: 0.57-0.78) were good (Table II).

**Table I:** Intraobserver agreement for absolute displacement, relative displacement, and vertical displacement (ICC and Gwet's AC1) overall and per variable (patient positioning, position of arm, and projection)

Variable	Overall	ICC (95% CI)
**Absolute displacement**		0.91 (0.87-0.93)
Patient positioning	Supine	0.87 (0.81-0.91)
	Upright	0.93 (0.89-0.96)
Positioning arm	Neutral	0.91 (0.86-0.94)
	Protracted	0.90 (0.84-0.94)
Direction X-ray beam	15° caudocranial	0.92 (0.88-0.95)
	15° craniocaudal	0.88 (0.83-0.92)
**Relative displacement**		0.92 (0.89-0.94)
Patient positioning	Supine	0.89 (0.83-0.93)
	Upright	0.94 (0.89-0.96)
Positioning arm	Neutral	0.92 (0.88-0.95)
	Protracted	0.92 (0.86-0.95)
Direction X-ray beam	15° caudocranial	0.93 (0.90-0.96)
	15° craniocaudal	0.90 (0.84-0.94)
		Gwet's AC1
**Vertical displacement**		0.77 (0.69-0.85)
Patient positioning	Supine	0.70 (0.57-0.82)
	Upright	0.86 (0.77-0.95)
Positioning arm	Neutral	0.78 (0.67-0.90)
	Protracted	0.77 (0.65-0.88)
Direction X-ray beam	15° caudocranial	0.78 (0.67-0.89)
	15° craniocaudal	0.78 (0.67-0.89)

*ICC*, intraclass correlation coefficient; *CI*, confidence interval.

**Table II:** Interobserver agreement for absolute displacement, relative displacement, and vertical displacement (ICC and Gwet's AC1) overall and per variable (patient positioning, position of arm, and projection)

Variable	Overall	ICC (95% CI)
**Absolute displacement**		0.67 (0.50-0.77)
Patient positioning	Supine	0.60 (0.43-0.73)
	Upright	0.69 (0.45-0.82)
Positioning arm	Neutral	0.67 (0.50-0.78)
	Protracted	0.66 (0.46-0.79)
Direction X-ray beam	15° caudocranial	0.70 (0.34-0.84)
	15° craniocaudal	0.63 (0.48-0.74)
**Relative displacement**		0.72 (0.56-0.81)
Patient positioning	Supine	0.65 (0.49-0.76)
	Upright	0.74 (0.51-0.85)
Positioning arm	Neutral	0.72 (0.56-0.82)
	Protracted	0.71 (0.51-0.83)
Direction X-ray beam	15° caudocranial	0.75 (0.39-0.87)
	15° craniocaudal	0.68 (0.54-0.78)
		Gwet's AC1
**Vertical displacement**		0.67 (0.57-0.78)
Patient positioning	Supine	0.53 (0.38-0.68)
	Upright	0.81 (0.71-0.92)
Positioning arm	Neutral	0.68 (0.55-0.81)
	Protracted	0.65 (0.52-0.79)
Direction X-ray beam	15° caudocranial	0.64 (0.51-0.78)
	15° craniocaudal	0.71 (0.59-0.84)

*ICC*, intraclass correlation coefficient; *CI*, confidence interval.

The measured average absolute (11.7 mm; SD, 9.5) and relative (7.9%; SD, 6.2) shortenings were found to be the smallest in the supine patient positioning with the arm in neutral orientation on a 15° caudocranial projection. This scenario was also the one resulting in the least vertical displacement (median, 100%; interquartile range, 0%-50% to 50%-100%). The average absolute (17.7 mm; SD, 10.2) and relative (11.9%; SD, 6.6) shortenings (17.7 mm, 11.9%) were found to be the greatest in the upright patient positioning with the arm protracted orientation on a 15° caudocranial projection. This scenario also was the one resulting in the most vertical displacement (median, >100%; interquartile range, >100% to >100%).

As for the individual variables, the average difference in results of measurements of absolute shortening when evaluating the influence of patient positioning between supine (12.9 mm; SD, 8.7) and upright (17.4 mm; SD, 9.1, range, 0-38) positioning was 4.5 mm (95% CI: 3.0-5.9, *P* < .0001). The difference in relative shortening between supine (8.5%; SD, 5.5) and upright (11.7%; SD, 5.8) positioning was 3.2% (95% CI: 2.2-4.1, *P* < .0001) (Table III). In the upright patient position, the odds of being scored a category higher were 4.7 (95% CI: 2.2-9.8) times as large as the odds of being scored a category higher in supine position when all other variables in the model were held constant (Table IV).

**Table III:** Results of measurements for absolute and relative shortening per variable including the differences per variable (largest measurement minus smallest measurement)

Variable	Mean (mm)	SD	Difference (mm) (95% CI)	*P* value
**Absolute shortening**				
Patient positioning				
Supine	12.9	8.7	4.5 (3.0 to 5.9)	<.0001
Upright	17.4	9.1		
Positioning arm				
Neutral	14.8	9.4	0.7 (−0.8 to 2.2)	.84
Protracted	15.5	9.0		
Direction X-ray beam				
15° caudocranial	15.2	9.9	0.1(−1.3 to 1.6)	.36
15° craniocaudal	15.1	8.5		
**Relative shortening**				
Patient positioning				
Supine	8.5	5.5	3.2 (2.2 to 4.1)	<.0001
Upright	11.7	5.8		
Positioning arm				
Neutral	9.9	6.0	0.4 (−0.5 to 1.3)	.42
Protracted	10.3	5.7		
Direction X-ray beam				
15° caudocranial	10.3	6.4	0.4 (−0.5 to 1.3)	.42
15° craniocaudal	9.9	8.5		

*SD*, standard deviation; *CI*, confidence interval.

**Table IV:** Proportional odds ratios and 95% confidence intervals of increasing a category (0%-50%, 50%-100%, >100%) in vertical displacement per variable

Variable	OR (95% CI)	*P* value
Supine: upright	4.7 (2.2-9.8)	<.0001
Neutral: protracted	1.0 (0.5-1.9)	.95
15° craniocaudal: 15°caudocranial	5.9 (2.1-12.6)	<.0001

*OR*, odds ratio; *CI*, confidence interval.

No statistically significant differences were found for either absolute (0.7 mm, 95% CI: −0.8 to 2.2) or relative shortening (0.4, 95% CI: −0.5 to 1.3), and vertical displacement (OR, 1.0; 95% CI: 0.5-1.9) concerning a change in position of the arm between neutral and protracted (Tables III and IV). No statistically significant differences in measurements were found when evaluating the influence of X-ray projection on both absolute and relative shortenings (Table III). However, the odds of being scored a category higher on the caudocranial projection were 5.9 (95% CI: 2.8-12.6) times as large as the odds of being scored a category higher on the craniocaudal projection when all other variables in the model were held constant (Table IV).

## Discussion

In the present study, we aimed to quantify the differences in measured shortening by using a standardized method of measuring displaced midshaft clavicle fractures in varying patient positions (supine vs. upright and arm in neutral vs. protracted position) and direction of the X-ray beam (15° caudocranial vs. 15° craniocaudal). We found a statistically significant difference in average measurements of absolute shortening using a standardized method of 4.5 mm between the supine and upright views when keeping all other variables constant. This difference is in line with Malik et al,^23^ who report a measured absolute shortening of −0.41 mm (95% CI: −2.53 to 1.70 mm) and 4.86 mm (95% CI: 1.66-8.06 mm) in supine and upright patient positioning, respectively—a difference of 5.27 mm. We also found a statistically significant difference in relative shortening between supine and upright patient positioning of 3.2%. Because De Giorgi et al^7^ predict an increase of failure in conservatively managed midshaft clavicle fractures that are shortened >9.8%, the differences measured in the present study between patient positions (8.5%; SD, 5.5 for supine vs. 11.7%; SD, 5.8 for upright) may be relevant in the decision-making algorithm.

Differences in orientation of the arm during imaging (neutral vs. protracted) did not result in either absolute or relative differences in measured shortening that may be of clinical relevance. It seems that the glenohumeral joint is mostly responsible for the difference in orientations of the arm evaluated and therefore do not translate into different positions of the fracture elements and thus do not influence the measured shortening. We did not calculate a statistically significant difference between the average absolute and relative shortening when evaluating the direction of the X-ray beam in 15° caudocranial and 15° craniocaudal views. This is different than what is reported in another study by our group in which we identified a clear and statistically significant difference between caudocranial and craniocaudal views.^16^ The fact that no difference was found here could be caused by inherent differences between DRRs and proper X-ray projections used in this study. The different projections are well controlled in DRRs, which may not be the case for proper X-rays. We used 15° caudocranial and 15° craniocaudal projections because this was found to be the smallest difference in between projections resulting in statistically significant differences.^16^ It is possible that by using larger angulations of projections (ie, 30° caudocranial and 30° craniocaudal views), a statistically significant and possibly clinically relevant difference could be identified.

As for vertical displacement, we found statistically significantly larger odds of 4.7 (95% CI: 2.2-9.8) to be scored a category higher between the supine and upright patient positioning. Multiple other authors^3^^,^^23^^,^^26^ also report an increase in vertical displacement between supine and upright patient positioning. Unlike the present study, they do not report these differences in categories but in absolute measurements. Backus et al^3^ report an average increase of vertical displacement of 7.5 mm comparing supine with upright radiographs. Malik et al^23^ found an increase in vertical displacement from 9.42 to 15.72 mm between the 2 patient positions. Lastly, Onizuka et al^26^ report an increase of 2.4 mm in vertical displacement. No statistically significant differences in vertical displacement were found between the different orientations of the arm.

A statistically significant difference was found when evaluating the caudocranial to craniocaudal projections for vertical displacement. A proportional odds ratio of 5.9 (95% CI: 2.8-12.6) was calculated for an increase in category. Caudocranial projections were scored in a higher category of vertical displacement more often. This is in line with the findings of Hoogervorst et al,^15^ who found an increase in choice for surgical management for caudocranial projections of the same fractured clavicle compared with its craniocaudal projections. Because shortening was found to be greater on the latter projections (craniocaudal), it was hypothesized that vertical displacement might have been larger on the caudocranial projection explaining the increased choice for surgical management.

Supine patient positioning with the arm in neutral orientation on a 15° caudocranial projection resulted in the smallest amount of shortening and vertical displacement.

Upright patient positioning with the arm in protracted orientation on a 15° caudocranial projection resulted in the largest amount of shortening and vertical displacement. In order to create comparable results based on shortening and vertical displacement of the midshaft clavicle fracture, it may be advised to report these measurements on an upright patient positioning on a 15° caudocranial projection irrespective of the orientation of the arm.

We found excellent intraobserver agreement in measurements of absolute shortening, relative shortening, and vertical displacement similar to those reported when using DRRs.^16^ Interobserver agreement for the 3 outcome measures was found to be good; however, agreement was lower than when DRRs were used.^16^

One of the strengths of this study is that multiple factors influencing the measurements on the fractured clavicle were evaluated in a clinically relevant manner. Another strength of this study is the use of a standardized method for measuring shortening and categorizing vertical displacement as proven by the good to excellent intra- and interobserver agreements.

A potential limitation of this study is that even though the protocol for the different patient positions, orientations of the arm, and X-ray beam direction was standardized, it was, unlike the use of DRRs, not a static condition. However, it is a good reflection of the process in clinical practice and therefore should not diminish its validity. Another limitation is the use of only the 15° caudocranial vs. 15° craniocaudal projections. Adding 30° angulated projections would have increased the radiation exposure to the participants in this study greatly. In a study more focused on the influence of projection in measurements of the fractured clavicle, this may be interesting to investigate.

The results of the present study can be used in further discerning the optimal imaging and measurement techniques of the fractured midshaft clavicle fracture.

## Conclusion

Absolute shortening, relative shortening, and vertical displacement were found to be the greatest in the upright patient positioning with the arm protracted orientation on a 15° caudocranial projection. There is a statistically significant and possibly clinically relevant difference in shortening of the same fractured midshaft clavicle between the supine and upright positions. No statistically significant differences were found for a change in position of the arm between neutral and protracted. Vertical displacement has statistically significant larger odds to be scored in a higher category for patient positioning and X-ray projection.

## Disclaimer

The authors, their immediate families, and any research foundations with which they are affiliated have not received any financial payments or other benefits from any commercial entity related to the subject of this article.
