# Supplementary material for: Influence of radiographic projection and patient positioning on shortening of the fractured clavicle
Source: JSES Int. 2020 May 18;4(3):503–7. doi: 10.1016/j.jseint.2020.03.005 (PMC7478989; doi:10.1016/j.jseint.2020.03.005)
Supplement: Supplementary Appendix S1 [file mmc1.docx]

**Supplement 1.** The 8 consecutive standardized and calibrated X-rays representing all possible combinations of the three evaluated variables (patient positioning, positioning of arm and projection).

1= AP 15° cranio-caudal clavicle radiograph in upright position with the palm of the ipsilateral hand on the contralateral ASIS.

2= AP 15° caudo-cranial clavicle radiograph in upright position with the palm of the ipsilateral hand on the contralateral ASIS.

3= AP 15° cranio-caudal clavicle radiograph in upright position with the arm in neutral position.

4= AP 15° caudo-cranial clavicle radiograph in upright position with the arm in neutral position.

5= AP 15° cranio-caudal clavicle radiograph in supine position with the palm of the ipsilateral hand on the contralateral ASIS.

6= AP 15° caudo-cranial clavicle radiograph in supine position with the palm of the ipsilateral hand on the contralateral ASIS.

7= AP 15° cranio-caudal clavicle radiograph in supine position with the arm in neutral position.

8= AP 15° caudo-cranial clavicle radiograph in supine position with the arm in neutral position.
